# A case report and literature review of neuropsychiatric lupus presenting with coma as the initial symptom

**DOI:** 10.1097/MD.0000000000047194

**Published:** 2026-01-16

**Authors:** Lei Pang, Xiaoyan Xu, Yifan Liu, Xiaodong Wang, Yingliang Wang

**Affiliations:** a Affiliated Hospital of Shandong Second Medical University, School of Clinical Medicine, Shandong Second Medical University, Weifang, Shandong Province, China; b Department of Rheumatology, Affiliated Hospital of Shandong Second Medical University, Weifang, Shandong Province, China.

**Keywords:** autoantibodies, coma, methylprednisolone pulse therapy, neuropsychiatric lupus, systemic lupus erythematosus

## Abstract

**Rationale:**

Neuropsychiatric lupus (NPSLE) represents a severe disease manifestation where early recognition significantly impacts prognosis. This report highlights the diagnostic challenges and management strategies in NPSLE presenting with coma, aiming to enhance clinical awareness of this life-threatening condition.

**Patient concerns:**

A 20-year-old female presented with acute coma of unknown origin. Initial evaluation revealed nonspecific cerebral edema on neuroimaging, while symptomatic treatment yielded minimal improvement. Physical examination showed gangrene of the right toes, indicating significant vascular compromise.

**Diagnoses:**

According to the 2019 EULAR management recommendations for systemic lupus erythematosus (SLE), this patient first meets the diagnostic criteria for SLE: positive antiphospholipid antibodies (antinuclear antibody), positive anti-double-stranded DNA antibodies, positive anti-Smith antibodies, decreased complement component 3 and complement component 4 levels, with a total classification criteria score ≥ 10 points, confirming the diagnosis of SLE. Subsequently, the patient developed neuropsychiatric symptoms as defined by the guidelines. After comprehensive exclusion of non-SLE related causes including infections, encephalitis, and metabolic abnormalities, combined with specific laboratory findings (the aforementioned autoantibodies and complement abnormalities) and imaging results, a final diagnosis of NPSLE was established.

**Interventions:**

Treatment comprised methylprednisolone pulse therapy (500 mg daily for 3 days) followed by maintenance corticosteroids, combined with intravenous immunoglobulin (25 mg daily for 5 days) and cyclophosphamide (400 mg weekly). Adjunctive measures included mannitol for cerebral edema management and appropriate analgesic therapy.

**Outcomes:**

The patient regained consciousness by day 5, followed commands despite residual right-sided weakness (grade 2). Significant motor improvement (grade 4 strength) occurred by day 12, supported by neuroimaging showing reduced cerebral lesions. Discharge occurred on day 18 with substantial neurological recovery, though digital necrosis persisted.

**Lessons:**

Unexplained coma in young females warrants consideration of autoimmune etiologies. Comprehensive immunological testing is crucial for accurate diagnosis. The combination of methylprednisolone, immunoglobulin, and cyclophosphamide demonstrated significant efficacy in severe NPSLE. Early recognition and aggressive immunosuppressive therapy are vital for optimizing outcomes.

## 1. Introduction

Systemic lupus erythematosus (SLE) is a systemic disease characterized by organ involvement, wherein various pathogenic autoantibodies and immune complexes contribute to the onset and progression of the disease.^[1]^ SLE manifests in a diverse range of clinical phenotypes, from mild mucocutaneous symptoms to visceral involvement. The involvement of vital organs is closely related to patient prognosis, and severe neurological involvement often leads to poor outcomes, necessitating aggressive diagnosis and treatment strategies. Neurological involvement in patients with SLE is termed neuropsychiatric lupus (NPSLE). It typically occurs during the acute or terminal stages of the disease and may affect the central nervous system (CNS) and/or the peripheral nervous system. The most prevalent CNS manifestation is an intractable headache, which is reported in >50% of cases. Additionally, the presence of various psychiatric manifestations makes diagnosis challenging.

The clinical manifestations of NPSLE lack specificity and vary in severity, ranging from subtle cognitive dysfunction (i.e., in patients with mild disease and no obvious NPSLE manifestations^[2]^) to acute confusional states, psychosis, seizures, and stroke.^[3]^ This variability often leads to misdiagnosis and inappropriate treatment of the disease. Severe NPSLE can be life-threatening. Zirkzee et al found that the survival rates of patients with NPSLE have not improved over the past few decades. However, early diagnosis and broader use of antiplatelet therapy may lead to improved outcomes.^[4]^ Therefore, early recognition of NPSLE is of paramount clinical importance. NPSLE presenting with coma as the initial symptom has rarely been reported. The inability to communicate with the patient often leads to misdiagnosis or delayed diagnosis in such cases, which can significantly affect the prognosis. In this report, we present a case of NPSLE in which coma was the initial manifestation. The patient achieved a favorable outcome following active treatment.

## 2. Case presentation

This study has been approved by the Ethics Committee of the Affiliated Hospital of Shandong Second Medical University (Approval No.: SDSMU-2025-qt-116). Written informed consent was obtained from the patient for the use of clinical data in this case report, and the study conforms to the ethical guidelines of the Declaration of Helsinki.

### 2.1. Chief complaint & present illness

**Figure 1.**
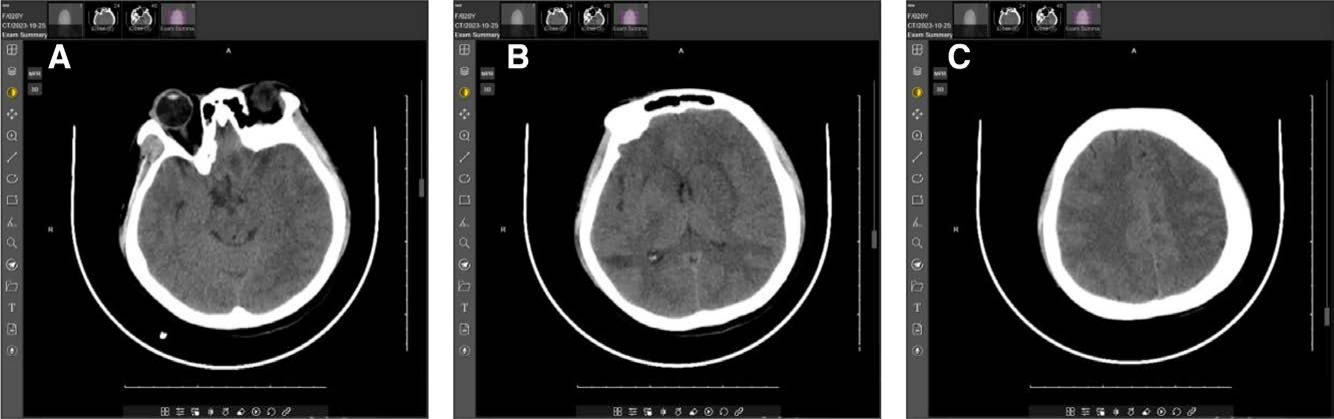
A-1C: October 25, 2023 – CT reveals multiple patchy hypodensities with ill-defined margins in the right frontal and temporal lobes, and bilateral basal ganglia. CT = computed tomography.

A 20-year-old woman experienced sudden coma without obvious triggers at 8:00 am on October 25, 2023. She opened her eyes to mild stimulation but remained unresponsive to verbal calls, with occasional spontaneous limb movements and urinary incontinence. Symptoms persisted without relief. Initial cranial computed tomography revealed cerebral edema (Fig. 1), leading to hospitalization for “coma of unknown cause.”

### 2.2. Past medical history & review of systems

The patient had been previously healthy. Two months earlier, she developed purplish discoloration of the right toes, which recently progressed to black necrosis. She sought no medical attention for these symptoms. Additional findings included limb ischemia, significantly elevated erythrocyte sedimentation rate (ESR), and pulmonary hypertension.

### 2.3. Physical examination on admission

The patient was comatose. Cardiopulmonary and abdominal examinations were unremarkable. Both lower limbs showed reduced skin temperature, particularly in the feet, with poor peripheral perfusion. The right foot exhibited purplish discoloration of the first to fourth toes and black necrosis of the fifth toe, without exudate.

### 2.4. Diagnostic investigations

#### 2.4.1. Neuroimaging

Computed tomography: Cerebral edema.

**Figure 2.**
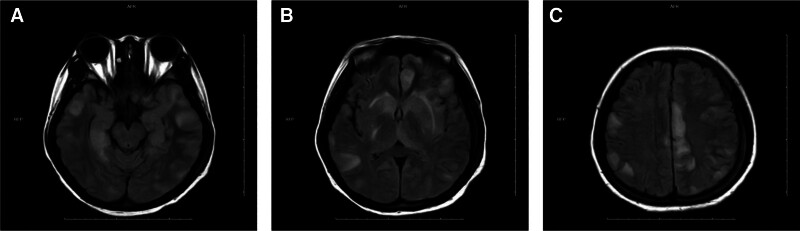
(A–C) October 27, 2023 – MRI demonstrates bilateral swelling with T2/FLAIR hyperintensity in the hippocampal formations, occipital lobes, temporal lobes, basal ganglia, frontal lobes, and parietal gyri. MRI = magnetic resonance imaging.

Magnetic resonance imaging (MRI): Swelling in bilateral frontal, parietal, and temporal lobes, hippocampus, left insular cortex, and basal ganglia; increased diffusion-weighted imaging signal in left temporal lobe, insula, and thalamus; short T2 signals near the left frontal-parietal midline (Fig. 2).

Magnetic resonance angiography (MRA): Stenosis in the distal right vertebral artery and origin of the right anterior cerebral artery.

Contrast MRI: Multiple bilateral cerebral lesions with leptomeningeal enhancement, suggesting encephalitis; thin, irregular venous sinuses indicating slow flow.

#### 2.4.2. Laboratory and ultrasound tests

Cerebrospinal fluid (CSF): Elevated protein (1.05 g/L), mononuclear cells (46%), and immunoglobulins; no bacteria or tumor cells.

Serology: Positive antinuclear antibody (1:3200, speckled), elevated anti-Smith, anti-double-stranded DNA antibody, and other SLE-specific antibodies.

Blood: Elevated inflammatory markers (ESR 56 mm/h, C-reactive protein 28.84 mg/L), anemia (hemoglobin 95 g/L).

Echocardiography: Mild tricuspid regurgitation and moderate pulmonary hypertension.

The section above highlights several laboratory investigations of significant clinical importance. The detailed results and reference ranges for all tested items have been compiled in Table 1.

**Table 1:** Laboratory parameters.

Test category	Specific test	October 25, 2023	October 26, 2023	October 27, 2023	October 28, 2023	October 29, 2023	November 4, 2023	November 10, 2023	November 12, 2023	Reference range
Test category	Specific test	October 25, 2023	October 26, 2023	October 27, 2023	October 28, 2023	October 29, 2023	November 04, 2023	November 10, 2023	November 12, 2023	Reference range
Coagulation profile	Fibrinogen (g/L)	4.14				2.74		3.05		2–4
	D-dimer (mg/L)	6.13				4.45		6.72		0–0.5
Liver & kidney function, glucose, electrolytes	Albumin (g/L)	35.8			29	30.2		32.3		40–55
	Globulin (g/L)	48			55.2	36.7		30.5		20–40
	Glucose (mmol/L)	4.02			8.45			4.46		3.8–6.1
	Uric acid (μmol/L)	570			189	247		324		135–425
	Calcium (mmol/L)	2.25						2.03	2.23	2.11–2.52
	Sodium (mmol/L)	135.8						133.1	141.4	137–147
	Alanine aminotransferase (ALT) (U/L)	10			22	24		177		7–40
	Aspartate aminotransferase (AST) (U/L)	21			16	15		178		13–35
	Gamma-glutamyl transferase (GGT) (U/L)	32			36	46		414		7–45
Complete blood count	White blood cell count (×10^9^/L)	15.05			5.21	17.81		5.49		3.5–9.5
	Neutrophil Count (×10^9^/L)	14.1			4.63	17.3		4.74		1.8–6.3
	Hemoglobin (g/L)	92			86	94		84		115–150
Serum amyloid A	Serum amyloid A (mg/L)		456.39					188.33		0–10
Procalcitonin	Procalcitonin (ng/mL)		0.108					0.669		<0.046
Thyroid function	Free T3 (pmol/L)		2.81							3.1–6.8
Erythrocyte sedimentation rate	ESR (mm/h)		70		88					0–20
C-reactive protein	CRP (mg/L)		75.9		7.26			32.9		0–5
Antinuclear antibody profile	ANA titer			1:3200 (S)	1:1000 (S)					<1:100
	Anti-SSA/60 kD (AU/mL)			847						0–120
	Anti-SSB (AU/mL)			207						0–120
	Anti-Smith (AU/mL)			1208						0–120
	Anti-nucleosome (AU/mL)			403						0–120
	Anti-dsDNA (AU/mL)			806						0–120
	Anti-ribosomal P (AU/mL)			378						0–120
	Anti-Ro-52 (AU/mL)			257						0–120
	Anti-histone (AU/mL)			506						0–120
	Anti-mitochondrial M2 (AU/mL)			374						0–120
Cerebrospinal fluid analysis	Lactate dehydrogenase (U/L)			58						10–25
	Glucose (mmol/L)			3.12						2.5–4.45
	Chloride (mmol/L)			127.3						120–130
	Adenosine deaminase (U/L)			1.6						0–40
	Protein (g/L)			1.05						0–0.4
	Color			Colorless						
	Transparency			Slightly Turbid						
	Clot/Film			No Clot						
	Protein qualitative			Negative						
	Cell count (×10^6^/L)			300						
	Mononuclear cells (%)			46						
	Polymorphonuclear cells (%)			54						
ToRCH panel	Rubella virus IgG (AU/mL)			8.221						<2
	Cytomegalovirus IgG (AU/mL)			5.96						<2
	HSV type I/II IgG (AU/mL)			19.94						<2
	Toxoplasma IgG (AU/mL)			3.76						<2
CSF immunoglobulins	CSF IgG (mg/L)				114					0–34
	CSF IgA (mg/L)				26.8					0–5
	CSF IgM (mg/L)				13.2					0–1.3
N-terminal pro-B-type natriuretic peptide	NT-proBNP (pg/mL)				1599					0–125
24-h urine protein	Protein (g/L)								0.104	0–0.1
Antiphospholipid antibodies	Anti-β2-glycoprotein I IgM (RU/mL)			10.2						<20
	Anti-β2-glycoprotein I IgA (RU/mL)			8						<20
	Anti-β2-glycoprotein I IgG (RU/mL)			6.3						<20
	Anti-cardiolipin IgM (U/mL)			6.5						≤12
	Anti-cardiolipin IgA (U/mL)			5.3						≤12
	Anti-cardiolipin IgG (U/mL)			11.1						≤12
	Lupus anticoagulant test			Negative						Negative
Ferritin	Ferritin (ng/mL)	57.6								13–150
Complement	C3 (g/L)	0.25								0.9–1.8
	C4 (g/L)	0.07								0.1–0.4
CSF autoimmune encephalitis Abs	Anti-NMDAR IgG				Negative					Negative
	Anti-AMPAR1 IgG				Negative					Negative
	Anti-AMPAR2 IgG				Negative					Negative
	Anti-GABABR IgG				Negative					Negative
	Anti-LGI1 IgG				Negative					Negative
	Anti-CASPR2 IgG				Negative					Negative

ANA = antinuclear antibody, C3 = complement component 3, C4 = complement component 4, CRP = C-reactive protein, ESR = erythrocyte sedimentation rate.

### 2.5. Diagnosis

According to the 2019 EULAR/ACR criteria scoring system, the patient meets the following criteria: Neuropsychiatric symptoms: 3 points; Antiphospholipid antibodies: 2 points; Anti-dsDNA antibody positive: 6 points; Anti-Smith antibody positive: 6 points; Low complement component 3 and low complement component 4: 4 points. A score of 10 or higher is sufficient for a diagnosis of SLE. With a total score of 21 points, the diagnosis of SLE is confirmed. NPSLE, based on limb ischemia, coma, characteristic MRI lesions, CSF abnormalities, and highly specific autoantibodies.

### 2.6. Treatment

Immunosuppressive therapy: Methylprednisolone pulse (500 mg IV, 3 days), maintenance (80 mg IV daily), intravenous immunoglobulin (IVIG) (25 mg/day, 5 days), and cyclophosphamide (400 mg weekly).

Supportive care: Mannitol for cerebral edema; celecoxib for foot pain.

### 2.7. Clinical course

Day 5: Regained consciousness, followed simple commands. Right-sided weakness (grade 2) persisted, with left limb strength at grade 4+. All right toes were necrotic.

**Figure 3.**
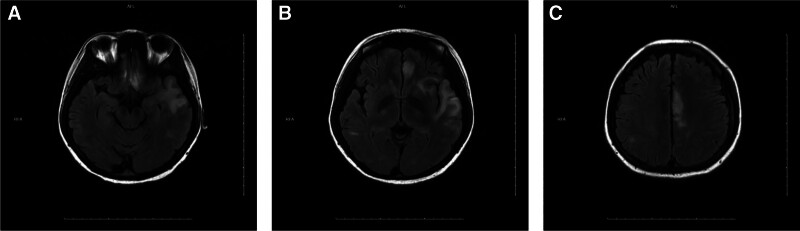
(A–C) November 7, 2023 – MRI reveals patchy areas of T2/FLAIR hyperintensity in the bilateral temporal lobes, basal ganglia, frontal lobes, and parietal lobes. MRI = magnetic resonance imaging.

Day 12: Significant improvement in right limb strength (grade 4). Right foot necrosis remained unchanged. Serial MRI showed reduced lesion volume (Fig. 3).

**Figure 4.**
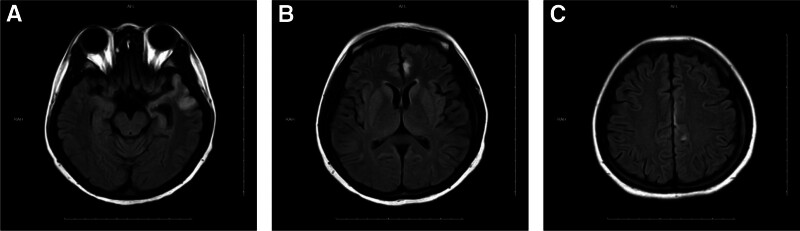
(A–C) December 7, 2023 – MRI demonstrates patchy T2/FLAIR hyperintensities in the left temporal, frontal, and parietal lobes. MRI = magnetic resonance imaging.

**Figure 5.**
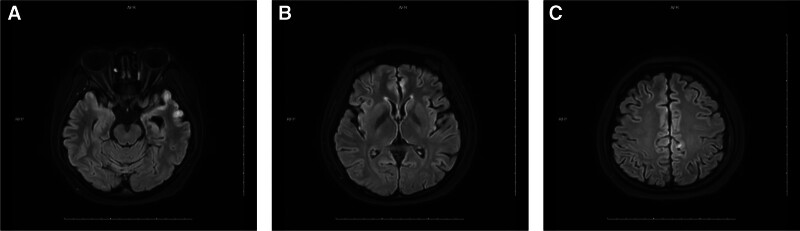
(A–C) March 1, 2024 – MRI demonstrates patchy T2/FLAIR hyperintensities in the left temporal and parietal lobes. MRI = magnetic resonance imaging.

Day 18: Stable and improved, discharged with planned brain MRI follow-up at 1 and 3 months (Figs. 4 and 5).

## 3. Discussion

The sudden onset of coma can be attributed to various medical conditions, including cerebrovascular, cardiac, hepatic, and renal diseases, hysteria, hypoglycemia, hypotension, and electrolyte imbalances. To administer targeted treatment, it is of paramount importance to promptly ascertain the cause of the coma. For instance, hypoglycemic coma in patients with diabetes is considered a diabetic emergency and is often associated with a history of diabetes and an excessive dosage of glucose-lowering medications. If not treated promptly, it can lead to irreversible brain damage and death in severe cases. Comas resulting from conditions such as cerebral hemorrhage, liver diseases, electrolyte imbalances, and cardiac pathologies are relatively easier to diagnose, and targeted treatments can lead to improvement. After completing the relevant examinations, the aforementioned diseases were ruled out. The patient presented with sudden onset of coma, and cranial MRI indicated the presence of cerebral edema, with lesions located in the bilateral cerebral hemispheres and basal ganglia. In addition, T2 signal abnormalities were observed in the frontal and parietal lobes, suggesting encephalitis-like changes. The possibility of intracranial lesions caused by viral, bacterial, or fungal infections leading to encephalitis was considered. However, the patient did not exhibit common signs of infection such as fever or elevated white blood cell count, and no pathogenic microorganisms were detected in the CSF examination. Physical examination revealed gangrene in the patient’s right foot, suggesting the potential for infection-induced gangrene in the right foot, which may have led to subsequent bloodstream infection by pathogenic microorganisms, leading to encephalitis. However, after completing the relevant diagnostic tests, infectious etiologies were largely ruled out. The patient was a young woman who presented with pulmonary hypertension, ischemic limb lesions, elevated ESR, and increased CSF protein levels. Based on these findings, the possibility of an autoimmune disease was considered. Further tests, including blood tests for autoantibodies and CSF immunoglobulin levels, were also performed. Combined with the cranial MRI findings, there was a high suspicion of “SLE, NPSLE, and lupus vasculitis.” After the patient regained consciousness, a history of “immune thrombocytopenia” was revealed, and it was noted that she had previously been treated with glucocorticoids, which led to some improvement in her condition. This finding further supported the diagnosis of “SLE.” Treatment with glucocorticoids, IVIG, and cyclophosphamide was initiated, resulting in rapid clinical improvement, further corroborating the diagnosis.The clinical manifestations of NPSLE are categorized into CNS manifestations and peripheral nervous system manifestations,^[5]^ as outlined in Table 2.

**Table 2:** Prevalence of neuropsychiatric features in patients with SLE.

Neuropsychiatric features in patients with SLE	Prevalence	
Central nervous system	Neurological syndromes (focal)	
	Seizure disorder	7.0–20.0
	Aseptic meningitis	0.3–2.7
	Cerebrovascular disease	8.0–15.0
	Demyelinating syndromes	0.9–27.0
	Headache	12.2–28.3
	Myelopathy	0.9–3.9
	Movement disorders	9.0
	Neuropsychiatric syndrome (diffuse)	
	Anxiety disorders	6.4–40.0
	Psychosis	0.6–11.0
	Mood disorders	7.4–65.0
	Acute confusional state	0.9–7.0
	Cognitive dysfunction	6.6–80.0
Peripheral central nervous system	Neurological syndromes (focal)	
	Autonomic disorders	0.08–1.3
	Myasthenia gravis	0.2
	Polyneuropathy	1.5–5.4
	Guillian–Barre syndrome	0.08–1.20
	Plexopathy	/
	Mononeuropathy	0.9–6.9

SLE = systemic lupus erythematosus.

As illustrated in the chart, the probability of impaired consciousness is relatively low compared to other manifestations. The patient initially presented with coma, which is a diffuse CNS manifestation of lupus encephalopathy. Because the patient was in a comatose state and unable to provide a detailed medical history, diagnosis was particularly challenging. A thorough physical examination and exclusion of other potential diseases was necessary to confirm the diagnosis. Additionally, we conducted a literature search for cases with coma as the presenting symptom, identified similar cases, and compared them with the current case, as shown in Table 3.

**Table 3:** Retrospective analysis of diagnostic and therapeutic evolution in neuropsychiatric SLE (NPSLE) (1872–2023).

Feature	First reported case (Kaposi,^[6]^ 1872)	Modern understanding foundational period (Bennahum and Messner,^[8]^ 1975)	Contemporary practice case (2023)	Retrospective analysis: evolutionary significance
Clinical presentation				
Chief manifestation	Central nervous system involvement	Coma, seizures, psychosis, and other diverse neuropsychiatric symptoms systematically described	Coma accompanied by limb ischemia	Evolution in disease understanding: Progressed from recognizing CNS involvement, to systematically characterizing symptoms like coma, to precisely linking coma with vasculitis (limb ischemia), completing the clinical picture.
Demographics	Predominantly affects young women	Predominantly young females (88.8% in study cohort)	20-yr-old female	Confirmation of high-risk population: Over 150 yr of observation consistently identifies young females as the core susceptible group for SLE and NPSLE.
Diagnostic workup				
Key findings	Postmortem examination suggested possible cerebral vasculitis.	• Positive brain scan (functional imaging) • Decreased CSF C4/IgG (indirect immune evidence)	Antemortem MRI/MRA: • Cerebral edema & inflammation • Arterial stenosis • Leptomeningeal enhancement (in vivo precise visualization)	Revolution in diagnostic paradigm: Shift from postmortem speculation → living functional & indirect immune assessment → living high-resolution anatomical & vascular imaging, enabling precise, visual diagnosis of pathology.
Serology	Not available Only clinical symptoms, signs, and microscopic examination	• Anti-neuronal antibodies (exploratory finding) • ANA/anti-dsDNA (early technology)	Highly specific autoantibody profile: • ANA (1:3200) • Anti-dsDNA, Anti-Sm, Anti-ribosomal P	Evolution of diagnostic evidence: From none → exploratory and nonspecific → highly specific, high-titer antibody profiles, providing a solid foundation for rapid diagnosis and mechanistic understanding.
Treatment strategy	Symptomatic treatment	• High-dose corticosteroids (core breakthrough) • Exploratory use of immunosuppressants	Combined Immunosuppression: • Methylprednisolone pulse • IVIG • Cyclophosphamide (multi-targeted intensive regimen)	Fundamental shift in treatment strategy: From no effective options → single-drug breakthrough → multi-targeted, standardized intensive therapy, resulting in a qualitative leap in treatment initiative and efficacy.
Clinical outcome	Very high mortality rate	High mortality (but signs of improvement already seen with steroid use)	Significant neurological recovery	Historic reversal of prognosis: patient outcomes transformed from inevitable death → high mortality with emerging hope → potential for functional recovery, marking the transition of NPSLE from a terminal illness to a manageable critical condition.

ANA = antinuclear antibody, anti-dsDNA = anti-double-stranded DNA antibody, C4 = complement component 4, CNS = central nervous system, IVIG = intravenous immunoglobulin, MRA = magnetic resonance angiography, MRI = magnetic resonance imaging, NPSLE = neuropsychiatric lupus, SLE = systemic lupus erythematosus.

This table systematically delineates the evolution of NPSLE from 1872 to 2023. The diagnostic paradigm has progressed from the 19th century reliance on postmortem inference of cerebrovascular pathology,^[6,7]^ to the 1975 achievement of antemortem immunological diagnosis through brain scanning and CSF analysis,^[8]^ and subsequently to the 2023 capability for precise visualization via MRI/MRA coupled with specific autoantibody profiling. Therapeutically, management has advanced from purely symptomatic support through the breakthrough of corticosteroid therapy to contemporary multi-targeted combined immunosuppressive regimens. Consequently, patient outcomes have transformed from historically high mortality rates to significant neurological recovery now being achievable, marking the transition of NPSLE from a fatal disease to a manageable critical condition.

NPSLE imaging lacks specificity and often manifests as scattered, patchy, subcortical hypodense lesions in the unilateral or bilateral cerebral hemispheres. These lesions have blurred boundaries and are typically distributed in the parieto-occipital or temporo-parietal lobes. Mild enhancement is observed in the marginal areas on contrast imaging. Additionally, some studies have reported the presence of round or oval, well-defined, hypodense lesions in the brainstem and basal ganglia.^[5]^ Cranial MRI findings in this patient revealed swelling in the bilateral frontal, parietal, and temporal lobes, hippocampus, left insular cortex, and bilateral basal ganglia. Increased diffusion-weighted imaging signals were observed in the left temporal lobe, insula, and dorsal thalamus. Additionally, multiple lesions were observed in both cerebral hemispheres, along with diffuse leptomeningeal enhancement. Distinguishing these findings from neurological tumors is crucial, particularly in the case of gliomas,^[9]^ which are the most prevalent type of primary brain tumors, accounting for 81% of CNS malignancies. Gliomas typically originate from glial cells or their precursor cells and can differentiate into astrocytomas, oligodendrogliomas, ependymomas, or oligoastrocytomas.^[10]^ Gliomas can also present with symptoms, such as impaired consciousness, which may lead to misdiagnosis and inappropriate treatment. The imaging findings of gliomas typically include irregularly shaped hyperdense lesions, often located in the cerebral lobes. Hemorrhagic areas are frequently accompanied by necrotic foci, tumor masses, and calcifications, with most lesions being surrounded by edema. These characteristics are inconsistent with the imaging findings in our patient.

NPSLE must also be differentiated from various types of meningitis. Common types of meningitis include purulent, tuberculous, viral, and cryptococcal meningitis (CM). Purulent meningitis is a severe intracranial infection caused by pyogenic bacteria and is often accompanied by the formation of brain abscesses with a high mortality rate. Bacteria are usually detected in the CSF.^[11]^ Tuberculous meningitis is caused by *Mycobacterium tuberculosis* and is difficult to treat. It is one of the more severe forms of tuberculosis and has a high mortality rate in children.^[12]^ Tuberculous meningitis typically presents as a subacute disease with nonspecific symptoms lasting for several days or weeks (average: 5–30 days), including low-grade fever, malaise, headache, dizziness, vomiting, personality changes, and symptoms related to pulmonary tuberculosis (such as cough). Patients with advanced disease may exhibit more severe headaches, altered mental status, stroke, hydrocephalus, and cranial nerve lesions. Viral meningitis is caused by epidemic viruses, such as enteroviruses, herpesviruses, and influenza viruses. The positivity rate for detecting these viruses in CSF using next-generation sequencing is relatively high.^[13]^ CM^[14]^ is a type of meningitis caused by a fungal infection, with *Cryptococcus* spp. commonly found in pigeon droppings. CM is a brain disease that results from CNS infection. The clinical course of CM is variable, but typically presents as subacute meningoencephalitis, characterized by a slow initial progression lasting from several days to weeks, followed by gradual deterioration. NPSLE and various types of meningitis share similar clinical manifestations, including headache, drowsiness, and meningeal irritation, which are all CNS symptoms. However, they can be distinguished through CSF analyses. In NPSLE, CSF analysis often reveals elevated immunoglobulin levels and the presence of autoantibodies. Conversely, in tuberculous meningitis, *M. tuberculosis* is typically identified, while in CM, *Cryptococcus* species can be identified. In this patient, CSF examination did not reveal the presence of bacteria, *M. tuberculosis*, viruses, or *Cryptococcus*, thereby excluding the diagnosis of meningitis.

Having thoroughly reviewed the relevant literature, we have provided our additional comments below. Macrophage activation syndrome (MAS) is a critical and life-threatening differential diagnosis in SLE patients presenting with neuropsychiatric symptoms.^[15]^ MAS, a severe form of secondary hemophagocytic lymphohistiocytosis, is characterized by high-grade fever, hepatosplenomegaly, cytopenias, and liver dysfunction. When a lupus patient presents with acute encephalopathy symptoms (such as seizures, confusion, or coma), clinicians must maintain a high index of suspicion for MAS, as these neuropsychiatric manifestations can overlap with those of primary neuropsychiatric SLE. Key differentiating features include the characteristically extreme hyperferritinemia (often > 10,000ng/mL), elevated triglycerides, decreased fibrinogen, and the finding of hemophagocytosis in the bone marrow or other tissues. The patient’s ferritin and triglyceride levels were not elevated, while fibrinogen was increased, thus ruling out MAS.

The diverse clinical manifestations of NPSLE are associated with complex underlying mechanisms. The pathogenesis of NPSLE is multifactorial and involves various inflammatory cytokines, genetic factors, multiple autoantibodies, blood-brain barrier dysfunction, complement activation, and the formation of immune complexes. These factors collectively contribute to vascular pathology, cytotoxicity, and autoantibody-mediated neuronal damage.^[16]^ Certain autoantibodies, such as anti-phospholipid (aPL),^[17]^ anti-ribosomal P, and brain cytoplasmic ribonucleic acid antibodies, disrupt normal brain function. Numerous studies have demonstrated a relationship between aPL antibodies and focal neurological manifestations of NPSLE (e.g., headaches, strokes, and seizures) and diffuse neurological manifestations (including acute consciousness disorders), indicating that these autoantibodies have pathogenic effects beyond their prothrombotic properties. Additionally, blood-brain barrier dysfunction and vascular pathology may contribute to the development of NPSLE. Research on the cerebrovascular aspects of NPSLE pathogenesis remains relatively limited. Autopsy findings in patients with NPSLE have revealed that cerebral microvascular ischemia and thrombosis, non-inflammatory small-vessel lesions, focal vascular occlusion, and microhemorrhages are common pathological features, suggesting that CNS damage is related to cerebrovascular pathology. Furthermore, vasculitis is a rare cause of NPSLE.^[18]^

There is no unified treatment protocol for NPSLE; however, various therapeutic approaches have been employed, including high-dose corticosteroids, methylprednisolone pulse therapy, IVIG, plasma exchange, immunosuppressants such as cyclophosphamide, azathioprine, and mycophenolate mofetil, and biologics such as rituximab.^[19]^

Cyclophosphamide is the only drug that has been compared with steroid pulse therapy in randomized controlled trials and has been found to be more effective than methylprednisolone pulse therapy for treating acute, severe NPSLE.^[20]^ IVIG can be combined with methylprednisolone and cyclophosphamide for the treatment of NPSLE. Similarly, IVIG is also utilized in autoimmune encephalitis (AE),^[21]^ given the significant clinical overlap between NPSLE and AE – where autoantibody-mediated neuroinflammation serves as a key shared mechanism. We employed IVIG as a rapidly acting immunomodulatory agent, with mechanisms beneficial to both conditions, including: Neutralization of pathogenic autoantibodies (e.g., anti-NMDAR antibodies in AE or anti-ribosomal P antibodies in NPSLE) and idiotypic regulation of antibody production; Attenuation of complement-mediated damage and suppression of pro-inflammatory cytokines. These actions provide critical and timely immunomodulation during the therapeutic window before slower-acting agents like cyclophosphamide achieve their full effect. The use of IVIG is well-supported in the management of severe neuroinflammatory disorders. A recent comprehensive review further reinforces its role in modulating immune responses in autoimmune neurological diseases.^[22]^

According to the 2010 EULAR recommendations for the management of NPSLE and the updated 2019 EULAR guidelines for SLE management, glucocorticoids and immunosuppressants are suitable for patients with NPSLE with symptoms related to inflammatory or immune-mediated processes (e.g., psychosis, acute confusional state, and myelitis). Antiplatelet or anticoagulant therapy is recommended in cases associated with aPL antibodies. Other treatments included symptomatic and supportive care as required. Therefore, systemic treatments, including immunosuppressive and antiplatelet/anticoagulant therapies, are the mainstay of NPSLE management. According to the 2023 EULAR updated consensus,^[23]^ for neuropsychiatric manifestations of SLE (NPSLE) that are inflammatory or immune-mediated (such as psychosis, acute confusional state, and myelitis), the recommended treatment pathway should be initiated with glucocorticoids and requires the rapid introduction of immunosuppressive agents. The patient in this case presented with acute, severe inflammatory symptoms. Therefore, we strictly adhered to this treatment pathway by implementing a combined therapeutic regimen of methylprednisolone pulse therapy, IVIG, and cyclophosphamide, and achieved a favorable clinical response.

## 4. Conclusion

In cases where a patient presents with coma as the initial symptom, particularly young women, it is imperative to comprehensively evaluate all potential etiologies. By characterizing coma as a distinct clinical phenotype of severe NPSLE, we have demonstrated the crucial importance of incorporating autoimmune etiology into the differential diagnosis. Our research validates a targeted diagnostic pathway integrating advanced neuroimaging (MRI/MRA) with comprehensive autoantibody profiling. Most significantly, we have established that immediate initiation of combined corticosteroid and immunosuppressant therapy upon diagnosis can substantially alter the disease course and achieve significant neurological recovery, thereby setting a new standard of care for such critical cases.

## Author contributions

**Conceptualization:** Lei Pang, Xiaodong Wang, Yingliang Wang.

**Data curation:** Lei Pang, Xiaodong Wang, Yingliang Wang.

**Formal analysis:** Lei Pang, Xiaoyan Xu, Yifan Liu.

**Funding acquisition:** Xiaodong Wang, Yingliang Wang.

**Investigation:** Lei Pang, Xiaoyan Xu, Yifan Liu.

**Methodology:** Lei Pang, Xiaodong Wang, Yingliang Wang.

**Resources:** Xiaodong Wang, Yingliang Wang.

**Supervision:** Lei Pang, Yingliang Wang.

**Validation:** Lei Pang, Xiaoyan Xu, Yifan Liu, Xiaodong Wang, Yingliang Wang.

**Visualization:** Lei Pang, Xiaoyan Xu, Yifan Liu.

**Writing – original draft:** Lei Pang.

**Writing – review & editing:** Lei Pang, Xiaodong Wang, Yingliang Wang.
